# Paternal Expressed Gene 10 (PEG10) is decreased in early-onset preeclampsia

**DOI:** 10.1186/s12958-023-01116-3

**Published:** 2023-07-18

**Authors:** Lydia Baird, Ping Cannon, Manju Kandel, Tuong-Vi Nguyen, Anna Nguyen, Georgia Wong, Cíara Murphy, Fiona C. Brownfoot, Elif Kadife, Natalie J. Hannan, Stephen Tong, Lucy A. Bartho, Tu’uhevaha J. Kaitu’u-Lino

**Affiliations:** 1 The Department of Obstetrics and Gynaecology, Mercy Hospital for Women, University of Melbourne, 163 Studley Road, Heidelberg Victoria, 3084 Australia; 2 Mercy Perinatal, Mercy Hospital for Women, Victoria, Australia

**Keywords:** Placenta, PEG10, Hypoxia, Inflammation, Preeclampsia, TGF, Cytotrophoblast

## Abstract

**Background:**

Preeclampsia is a severe complication of pregnancy which is attributed to placental dysfunction. The retrotransposon, Paternal Expressed Gene 10 (PEG10) harbours critical placental functions pertaining to placental trophoblast cells. Limited evidence exists on whether PEG10 is involved in preeclampsia pathogenesis. This study characterised the expression and regulation of PEG10 in placentas from patients with early-onset preeclampsia compared to gestation-matched controls.

**Methods:**

PEG10 expression was measured in plasma and placentas collected from patients with early-onset preeclampsia (< 34 weeks’) and gestation-matched controls using ELISA (protein) and RT-qPCR (mRNA). First-trimester human trophoblast stem cells (hTSCs) were used for in vitro studies. *PEG10* expression was measured during hTSC differentiation and hTSC exposure to hypoxia (1% O_2_) and inflammatory cytokines (IL-6 and TNFα) using RT-qPCR. Functional studies used *PEG10* siRNA to measure the effect of reduced PEG10 on canonical TGF-$\beta$ signalling and proliferation using luciferase and xCELLigence assays, respectively.

**Results:**

*PEG10* mRNA expression was significantly reduced in placentas from patients with early-onset preeclampsia (< 34 weeks’ gestation) relative to controls (*p* = 0.04, *n* = 78 vs *n* = 18 controls). PEG10 protein expression was also reduced in preeclamptic placentas (*p* = 0.03, *n* = 5 vs *n* = 5 controls, blinded assessment of immunohistochemical staining), but neither PEG10 mRNA nor protein could be detected in maternal circulation. PEG10 was most highly expressed in hTSCs, and its expression was reduced as hTSCs differentiated into syncytiotrophoblasts (*p* < 0.0001) and extravillous trophoblasts (*p* < 0.001). Trophoblast differentiation was not altered when hTSCs were treated with PEG10 siRNA (*n* = 5 vs *n* = 5 controls).

*PEG10* was significantly reduced in hTSCs exposed to hypoxia (*p* < 0.01). *PEG10* was also reduced in hTSCs treated with the inflammatory cytokine TNF $\alpha$ (*p* < 0.01), but not IL-6. PEG10 knocked down (siRNA) in hTSCs showed reduced activation of the canonical TGF-β signalling effector, the SMAD binding element (*p* < 0.05) relative to controls. PEG10 knockdown in hTSCs however was not associated with any significant alterations in proliferation.

**Conclusions:**

Placental PEG10 is reduced in patients with early-onset preeclampsia. In vitro studies suggest that hypoxia and inflammation may contribute to PEG10 downregulation. Reduced PEG10 alters canonical TGF-$\beta$ signalling, and thus may be involved in trophoblast dysfunction associated with this pathway.

## Background

Preeclampsia complicates 2–8% of pregnancies and is associated with significant maternal and fetal morbidity [1, 2]. Clinically, it is characterised by widespread endothelial dysfunction, *de-novo* hypertension and organ damage [3]. Early-onset preeclampsia is defined as diagnosis prior to 34 weeks’ gestation, and is associated with impaired placentation [4]. Pathogenesis of the disease is often attributed to placental insufficiency from inadequate trophoblast invasion and remodelling of maternal spiral arteries [5, 6]. Placental hypoxia, inflammation and oxidative stress also ensues due to poor placental perfusion [6]. The precise aetiology of preeclampsia remains poorly understood despite being recognised since 400 BCE by Hippocrates [7, 8]. Investigating potential pathogenic molecules is imperative to identify novel diagnostic and therapeutic targets for preeclampsia to improve maternal and fetal health outcomes worldwide.

Evolution of the eutherian placenta correlates with genomic integration of retroviral-derived retrotransposons [9–12]. Paternal Expressed Gene 10 (PEG10) is one of three members of the sushi-ichi-related retrotransposon homolog family which are involved in early placentation [13]. PEG10 is highly expressed in the placenta and is critical for normal placental development [14]. Whole-gene knockout of PEG10 in mice demonstrate early embryonic lethality due to impaired placental development [15, 16]. PEG10 has also been shown to play a role in specifying trophoblast cell lineages, as deletion of the PEG10-RF1 domain impairs trophoblast differentiation [17]. It is likely that PEG10 has multiple functions within the placenta [16].

Over-expression of PEG10 is associated with tumorigenesis, particularly in hepatocellular and breast carcinoma [18–21]. Its expression is positively associated with cellular proliferation in several cancers [22–24]. Emerging evidence suggests an indirect interaction between the transforming growth factor beta (TGF-β) signalling pathway and PEG10 [18]. Downstream effectors of the canonical TGF-β pathway have been shown to be activated by PEG10 in adenocarcinomas [25]. TGF-β signalling also functions in cell fate and body axis patterning during early gestation [26]. However, there is limited evidence on whether PEG10 is involved in TGF-β signalling functions in placentation.

The expression of PEG10 in preeclampsia has been poorly explored and only two prior manuscripts have been published. Chen et al*.* showed increased PEG10 expression in late-onset preeclampsia while Liang et al*.* found reduced PEG10 in preeclamptic placentas [27, 28]. Neither of these studies reported on PEG10 expression in early-onset preeclampsia.

This study aimed to assess placental expression of PEG10 in patients with early-onset preeclampsia using gestation-matched controls. Next, i*n vitro* studies characterised the role of PEG10 in preeclampsia pathogenesis using human first-trimester cytotrophoblast stem cells.

## Methods

### Early onset preeclampsia < 34 weeks’ gestation

Placental tissue was collected from patients at Mercy Hospital for Women (Melbourne, Australia) with early-onset preeclampsia (< 34 weeks’ gestation, *n* = 79) and gestation-matched controls (< 34 weeks’ gestation, *n* = 18) who delivered via caesarean section. Early-onset preeclampsia was diagnosed in accordance with American College of Obstetricians and Gynaecologists (ACOG) guidelines [29]. Preterm control placentas were obtained from normotensive patients who delivered preterm due to complications such as placenta previa. Ethics approval was granted by Mercy Health Human Research Ethics Committee (R11/34) and participants presenting to the Mercy Hospital for Women gave informed, written consent for sample collection. Patient characteristics are provided in Table 1.

**Table 1:** Patient characteristics of placental samples obtained (< 34 weeks)

	**Controls (*n* = 18)**	**Preeclampsia (*n* = 79)**	***P*-value (Control vs preeclampsia)**
**Maternal Age** (years) Mean ± SEM	31.50 ± 1.64	30.75 ± 0.62	0.81
**Gestation at Delivery** (weeks) Mean ± SEM	30.25 ± 0.59	29.65 ± 0.27	0.68
**BMI** (kg/m^2^) Median (IQR)	28.20 (35.08–24.75)	27.00 (35.70–24.75)	> 0.99
**Parity** no. (%) 0 1 ≥ 2	4 = count (22.20 of whole) 9 (50.00) 5 (27.80)	54 (68.35) 17 (21.52) 8 (10.13)	0.0015
**SBP at Delivery** (mmHg) Median (IQR)	125.00 (130.00–117.3)	170.0 (180.00–160.00)	< 0.0001
**DBP at Delivery** (mmHg) Median (IQR)	75.00 (80.00–70.00)	102.50 (110.00–99.75)	< 0.0001
**Birth weight** (g) Median (IQR)	1587 (2011–1298)	1099 (1431–841)	0.0026
**Assigned female at birth** no. (%)	10 (55.56)	40 (50.63)	0.71

*BMI* Body mass index, *SBP* Systolic blood pressure and *DBP* Diastolic blood pressure. Ordinary one-way ANOVA test was used for normally distributed data, Kruskal–Wallis test was used for non-parametric, and Chi-square tests for categorical variables. BMI data missing for 4/18 control samples, 9/79 PE samples. SBP and DBP data missing for 1/79 PE samples. Birth Weight data missing for 1/79 PE samples

### Tissue and blood collection

Placental samples were collected immediately after delivery. Tissue was randomly sampled from four separate sites of the placenta, washed in ice-cold Dulbecco’s phosphate-buffered saline (dPBS) and preserved with RNAlater Stabilization Solution (InvitrogenTM, Waltham, USA). Samples were stored at –80 °C for RNA and protein extraction. Maternal peripheral whole blood (2.5 mL) was collected in PAXgene whole blood RNA tubes (PreAnalytix, Hombrechtikon, Switzerland) when patients attended the hospital for routine appointments. Tubes were stored at room temperature for 24–72 h as per manufacturer’s instructions before being transferred to -20 °C for 24 h and stored at -80 °C until RNA extraction.

### Immunohistochemistry

PEG10 was localised by immunohistochemistry (IHC) in placental tissue collected from patients with early-onset preeclampsia (*n* = 5) compared to pre-term controls (*n* = 5). Paraformaldehyde-fixed placental tissue Sects. (5 μm) were dewaxed in xylene twice and rehydrated in descending grades of ethanol (100%, 90%, 70%) for 3 min each (all chemicals were obtained from Sigma-Aldrich, unless otherwise stated). Sections underwent antigen retrieval using 0.01 M sodium citrate buffer (pH 6.0) for 20 min. Sections were washed in phosphate-buffered saline (pH 7.6). Sections were exposed to endogenous peroxidase quenching and blocking (Rabbit Specific HRP/DAB Detection IHC kit; Abcam, Cambridge, UK). Sections were incubated for 1 h at 37 °C with rabbit anti-human PEG10 (Sigma, St. Louis, USA) at a concentration of 1 µg/mL in 1% BSA/PBS. For isotype control, primary antibody was substituted with rabbit IgG (Dako, Glostrup, Denmark). Biotinylated goat anti-rabbit IgG(H + L) (Abcam, Cambridge, UK) was used as a secondary antibody. Sections were lightly counterstained with Haematoxylin (Mayer’s) Solution diluted 1:10 in distilled water, dehydrated, and mounted using DPX mounting medium (BDH Laboratory Supplies, Poole, England). PEG10 immunostaining within cytotrophoblast cells was scored for each placenta, by five individuals, blinded to clinical parameters. Immunostaining was assessed using a score of staining intensity (0 for no staining, 1 for low intensity, 2 for medium intensity, 3 for high intensity and 4 for maximum intensity). Staining was visualized and imaged using Nikon Eclipse Ci-L, Nikon Microscope Digital Camera DS-Fi3 and NIS-Elements 5.01.00 (Minato, Tokyo, Japan).

### In situ hybridisation

*PEG10* and platelet endothelial cell adhesion molecule (*PECAM-1*) RNA was detected by in situ hybridisation technique using RNAScopeTM 2.5 HD Duplex Assay (In Vitro Technologies, Melbourne, Australia) as per manufacturer’s instructions. In situ hybridisation was conducted on neutral buffered formalin-fixed placental tissue sections dewaxed in xylene for 10 min and rehydrated in 100% ethanol for 6 min. Probes were hybridised for human-PEG10 and human-PECAM1 (In Vitro Technologies, Melbourne, Australia) for 2 h at 40 °C. Slides were counterstained with 10% haematoxylin and briefly submerged in 0.02% ammonia water prior to imaging. Staining was visualized and imaged using Nikon Eclipse Ci-L, Nikon Microscope Digital Camera DS-Fi3 and NIS-Elements 5.01.00 (Minato, Tokyo, Japan).

### Culture and differentiation of human trophoblast stem cells

First trimester human trophoblast stem cell (hTSCs) lines were imported from the RIKEN BRC through the National BioResource Project of the MEXT/AMED, Japan. Cells were cultured and differentiated into syncytiotrophoblast or extravillous trophoblast cells according to the methods developed by Okae et al. [30]. Cells were incubated at 37 °C with 8% O_2_ (physiological normoxic conditions).

### hTSCs cultured under hypoxic conditions

Cells were seeded at 60,000 per well in a 24-well cell culture plate and incubated in either 1% oxygen (hypoxic conditions) or 8% oxygen (physiological normoxic conditions) for 48 h (triplicate, *n* = 5). Cell lysates were collected for RNA extraction and qRT-PCR.

### hTSCs exposed to tumour necrosis factor alpha (TNFα) or interleukin-6 (IL-6)

hTSCs were seeded at 40,000 cells per well in a 24-well plate and incubated overnight at 37 °C in 8% oxygen. Cells were treated (triplicate, *n* = 5) for 24 h with 0, 0.1, 1 and 10 ng/mL doses of TNFα (Life Technologies, Carlsbad, USA), IL-6 (RnD systems, Minnesota, USA), or vehicle control (sterilised water or dPBS respectively). Following treatment, cell lysates were collected for RNA extraction and qRT-PCR.

### siRNA knockdown of *PEG10 in-vitro*

hTSCs were transfected with either 50 nM *PEG10* (Horizon Discovery, Cambridge, UK) or negative control (Qiagen, Hilden, Germany) short interfering ribonucleic acid (siRNA) diluted in Opti-MEM medium (Life Technologies, Carlsbad, USA). siRNA was complexed with 1 pmol of Lipofectamine RNAiMAX Transfection Reagent (Invitrogen, Waltham, USA) for 20 min prior to dropwise addition to cells. Cells were incubated at 37 °C in 8% oxygen for 48 h with media replaced at 24 h.

### Luciferase assay

hTSCs were seeded at 12,500 cells/well in a 96-well white plate (Greiner Bio-one, Kremsmünster, Austria) and incubated overnight at 37 °C and 8% oxygen. Cells were treated with *PEG10* siRNA and incubated overnight. The plasmids, SBE4-Luc [31] (SBE4-Luc was a gift from Bert Vogelstein (Addgene plasmid # 16,495; http://n2t.net/addgene:16495; RRID:Addgene_16495) Addgene, Watertown, USA) and pSV-β- Galactosidase (Promega, Madison, USA) were prepared as a single colony inoculated in 250 mL of Gibco LB broth (Thermo Fisher Scientific, Waltham, USA) with ampicillin and grown overnight at 37 °C on a shaker. Plasmid DNA was extracted using the PureLink HiPure Plasmid Maxiprep kit (Thermo Fisher Scientific, Waltham, USA) according to the manufacturer’s instructions. Cells were treated with either 0.125 µg of SBE4-Luc, pSV-β- Galactosidase (used as a transfection control) or both. For control empty vector, Topo (Origene, Rockville, USA) was used. Lipofectamine 3000 Transfection Reagent (Invitrogen, Waltham, USA) was diluted 1:50 in Opti-MEM medium and complexed with plasmids for 20 min prior to dropwise treatment to cells. Following overnight incubation, the Dual-Luciferase® Reporter Assay System (Promega, Madison, USA) was used according to manufacturer’s instructions. Transfection efficiency was normalized with the co-transfected pSV-β-Galactosidase control vector.

### xCELLigence

xCELLigence plates (ELITechGroup, Paris, France) were coated overnight with 0.5 μg/cm^2^ iMatrix-511 (Matrixome, Japan) in PBS (Gibco, Australia) prior to washing and seeding with 6000 cells per well. Plates were incubated at 37 °C at 5% CO_2_ and 20% O_2_ in a real-time Cell Analyzer MP instrument (Roche Diagnostics, Basel, Switzerland). After 24 h, hTSCs were treated with *PEG10* siRNA using methods previously described, and the media was changed 24 h later, and experiment concluded 74 h post treatment. Impedance measurements were recorded every hour.

### Isolation and culture of primary term cytotrophoblast

Primary cytotrophoblast were isolated from normoxic term placentas using methods previously published [32]. After 24 h adherence under 8% Oxygen and 5% CO_2_, cells were either kept at 8% Oxygen (normoxia) or moved to 1% Oxygen (hypoxia) for 48 h before mRNA was isolated for gene analysis.

### Quantitative RT-PCR to measure *PEG10* gene expression

RNA was extracted from placental samples, cytotrophoblast, syncytiotrophoblast, and extravillous trophoblast cells using the GenEluteTM Mammalian Total RNA Miniprep Kit (Sigma-Aldrich, St. Louis, USA) following the manufacturer’s instructions. RNA was quantified using the NanoDropTM 2000 Spectrophotometer (Thermo Scientific, Waltham, USA). A total of 1 µg placental RNA or 100 ng cellular RNA were reverse transcribed to cDNA using the High-Capacity cDNA Reverse Transcription Kit (Life Technologies, Carlsbad, USA), as per the manufacturer’s instructions. RNA was converted to cDNA using the iCycler iQ5 (BioRad, Hercules, USA) following the manufacturer’s instructions. Taqman Fast Advanced Master Mix (ThermoFisher Scientific, Waltham, USA) and specific fluorescein amidite (FAM)-labelled Taqman gene expression assays (Life Technologies, Carlsbad, USA) were used to measure mRNA expression of *PEG10* (Assay ID: Hs01122880_m1), *CDH2* (n-cadherin 2, Assay ID: Hs00983056_m1), *GATA3* (GATA binding protein 3, Assay ID: Hs00231122_m1), *HLAG* (human leukocyte antigen G, Assay ID: Hs03045108_m1), *SDC1* (syndecan-1, Assay ID: Hs00896423_m1) and *TEAD4* (TEA domain transcription factor 4, Assay ID: Hs01125032_m1). Quantitative RT-PCR was performed on the CFX384 (BioRad, Hercules, USA) with the following conditions: 95 °C for 20 s, 40 cycles of denaturation for 95 °C for 3 s and 60 °C for 30 s. No product was detected in the non-template control and gene expression data was normalised to housekeeper genes: The geometric mean of *CYC-1* (Cytochrome c1, Assay ID: Hs00357717_m1) and *TOP1* (topisomerase 1, Assay ID: Hs00243257_m1) for placental samples. For cytotrophoblast and extravillous cytotrophoblast to *CYC-1,* and *GAPDH* (Glyceraldehyde 3-phosphate dehydrogenase, Assay ID: Hs99999905_m1) for syncytiotrophoblasts. Samples were run as duplicates and the mean quantification cycle (Cq) was used. Gene expression was normalised to the cycle threshold (Ct) mean of each control group, and the 2^−ΔΔCt^ method of the mean was used and expressed as fold change relatives to controls.

### Statistical analysis

Maternal characteristics were compared for women diagnosed with early onset preeclampsia, compared to normotensive, gestation-matched controls using a Mann–Whitney U test for continuous data and Fisher’s exact test for categorical data. *In-vitro* experiments were performed in technical duplicates and repeated at least four times. The data obtained was tested for normality using the Anderson–Darling test, D’Agostino & Pearson test, Shapiro–Wilk test, and Kolmogrov-Smirnov test. For two unpaired groups, an unpaired t-test (parametric) or Mann–Whitney test (non-parametric) test was used. For analysis of two paired groups, a paired t-test (parametric) or a Wilcoxon ranked test (non-parametric) was used. For ≥ 3 groups, either one-way ANOVA (parametric) or a Kruskal Wallis test (non-parametric) was used. Parametric data was represented as mean ± standard error of the mean (SEM) while non-parametric data was represented as a median with interquartile range (IQR). *P* < 0.05 were considered statistically significant. All analyses were performed using GraphPad Prism 9.4.0 (GraphPad Software, LLC.).

## Results

### *PEG10* gene expression is reduced in placenta from patients with early-onset preeclampsia

**Fig. 1:**
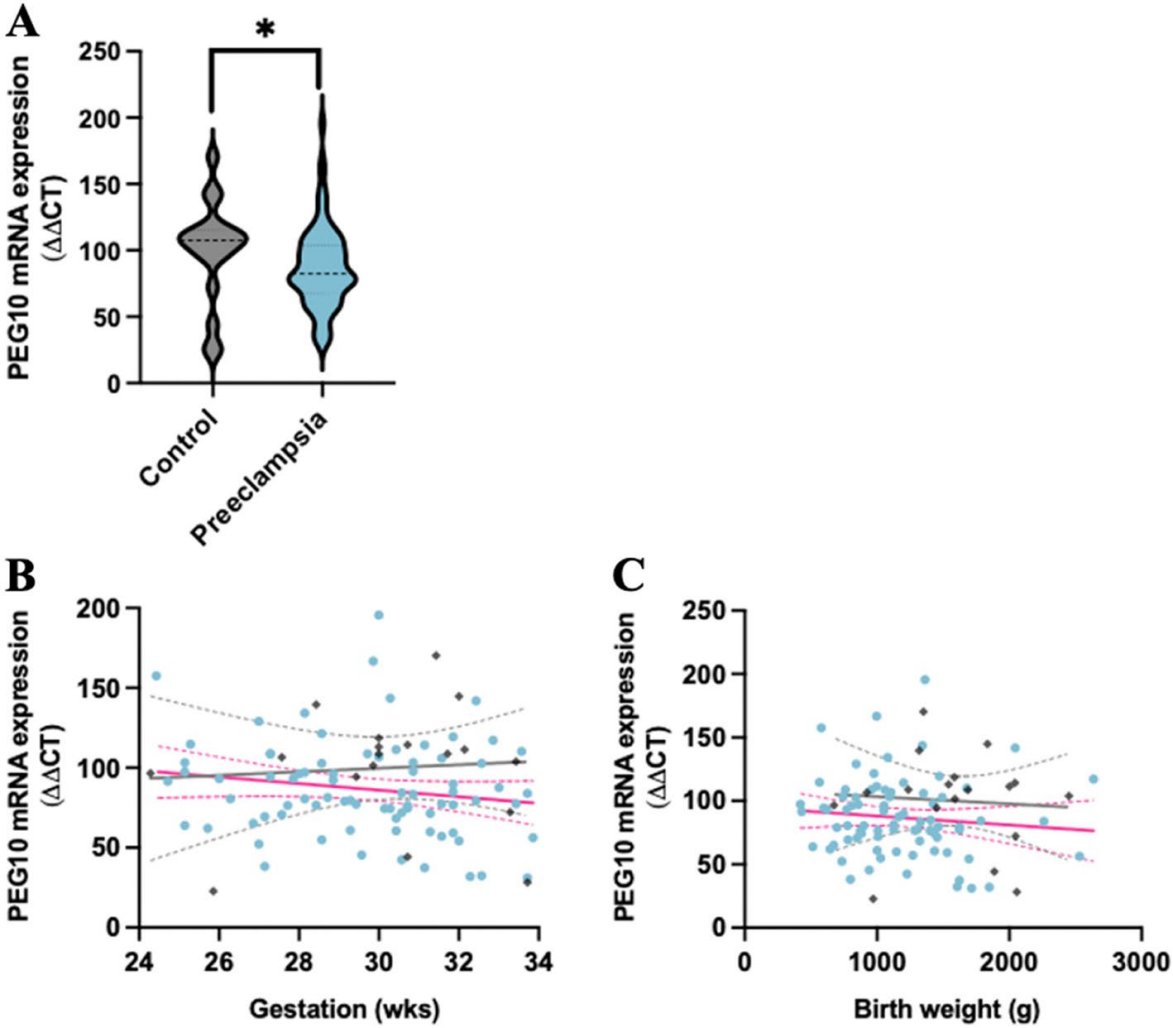
PEG10 mRNA expression in placental lysates from patients with early-onset preeclampsia. *PEG10* mRNA expression was reduced in placentas from 79 women with early-onset preeclampsia (pink line) compared to 18 gestation-matched preterm controls (grey) (**A**). Further analysis of the early-onset preeclampsia cohort (blue circle) and gestation-matched pre-term controls (grey diamond) showed no significant association between gestation (**B**) or birth weight (**C**). Data expressed as a median with interquartile range. Simple linear regression was used to present a line of best fit (solid line) and 95% confidence interval (dashed line). Individual symbols represent individual patients. P* < 0.05

*PEG10* gene expression was measured in placental lysates from 79 women with early-onset preeclampsia, compared to 18 gestation-matched controls. *PEG10* mRNA was significantly reduced in placenta from patients with early-onset preeclampsia, relative to controls (Fig. 1A, *P* = 0.04). Further analysis revealed no significant association between *PEG10* mRNA expression and gestation (Fig. 1B), fetal birth weight (Fig. 1C) or fetal sex (data not shown).

To assess the potential of PEG10 as a diagnostic biomarker for early-onset preeclampsia, PEG10 mRNA and protein was measured in maternal whole blood and plasma, respectively. Neither PEG10 mRNA nor protein could be detected in the maternal circulation at the time of sample collection.

### PEG10 protein and gene expression is localised to cytotrophoblasts

**Fig. 2:**
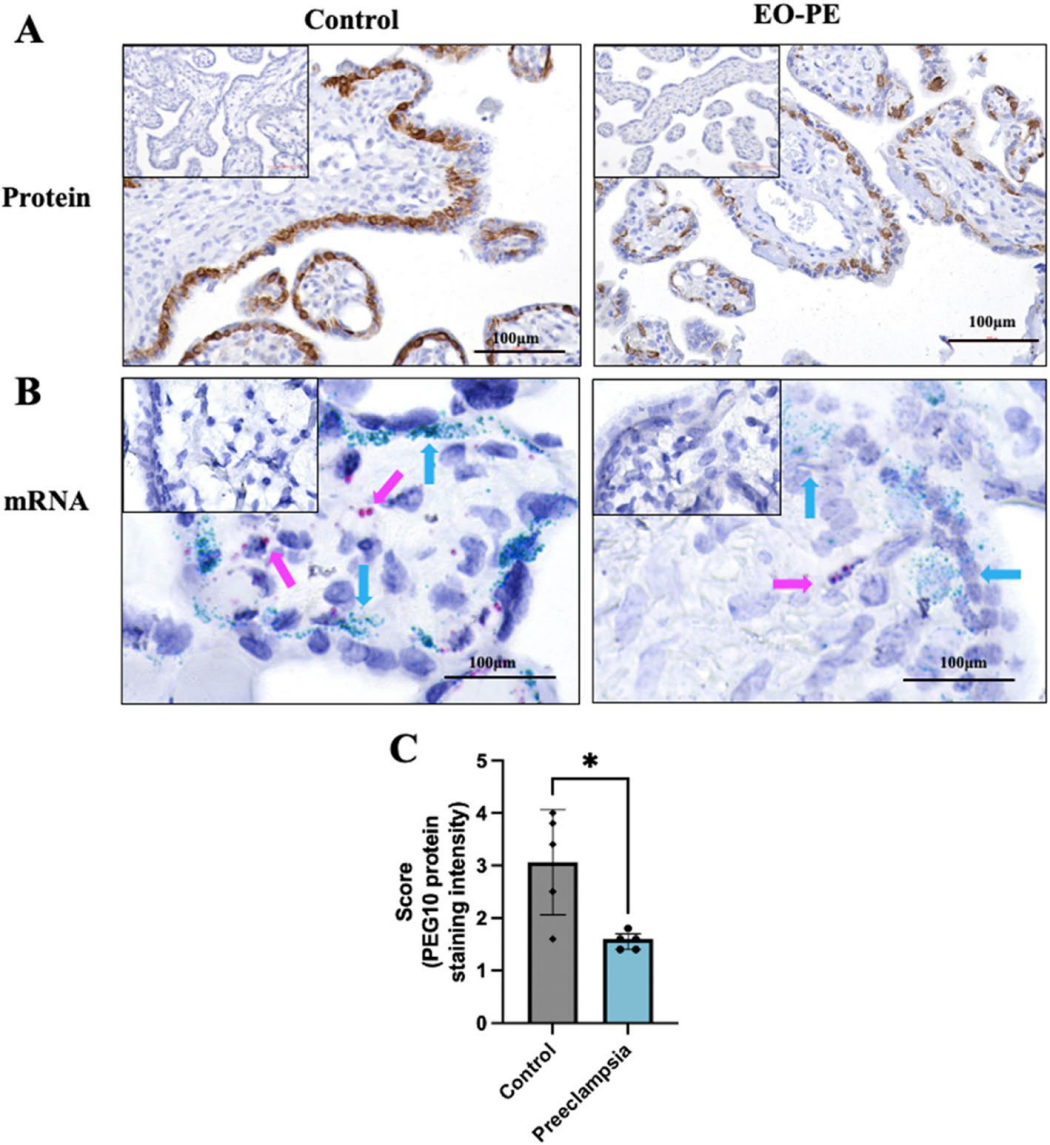
Localisation of PEG10 protein and mRNA within the placenta. Immunohistochemistry (IHC) staining of human placental villi shows PEG10 protein (brown) localised to cytotrophoblasts in both control and early-onset preeclampsia tissue (**A**). Semi-quantitative, blinded assessment of IHC staining indicated reduced PEG10 protein in early-onset preeclampsia placentas relative to controls (**C**). In situ hybridisation shows *PEG10* (blue) and *PECAM-1* (pink) mRNA expression. *PEG10* expression localised to cytotrophoblasts in both control and early-onset preeclampsia tissues while *PECAM-1* marked placental vasculature (**B**). No staining observed in respective isotype controls (minimised image). Figures were captured at 40 × magnification and are representative of 5 experimental repeats. Data expressed as mean $\pm$ SEM. Individual symbols represent individual scores and P* < 0.05

PEG10 localisation was assessed in the placenta via immunohistochemistry. Staining of placental sections confirmed PEG10 (brown) localisation to cytotrophoblast cells in tissues from control and preeclamptic pregnancies (Fig. 2A). Semi-quantitative blinded assessment of IHC staining by 5 individuals confirmed reduction of PEG10 in the cytotrophoblast from early-onset preeclampsia placentas (*n* = 5), compared to controls (*n* = 5, *P* = 0.03; Fig. 2C).

In situ hybridisation confirmed *PEG10* mRNA (blue) localisation to cytotrophoblasts. Staining was apparent in placenta from patients with either early-onset preeclampsia or controls (Fig. 2B). Platelet endothelial cell adhesion molecule (*PECAM-1*) was localised (pink) to detect placental vasculature (Fig. 2B).

**Fig. 3:**
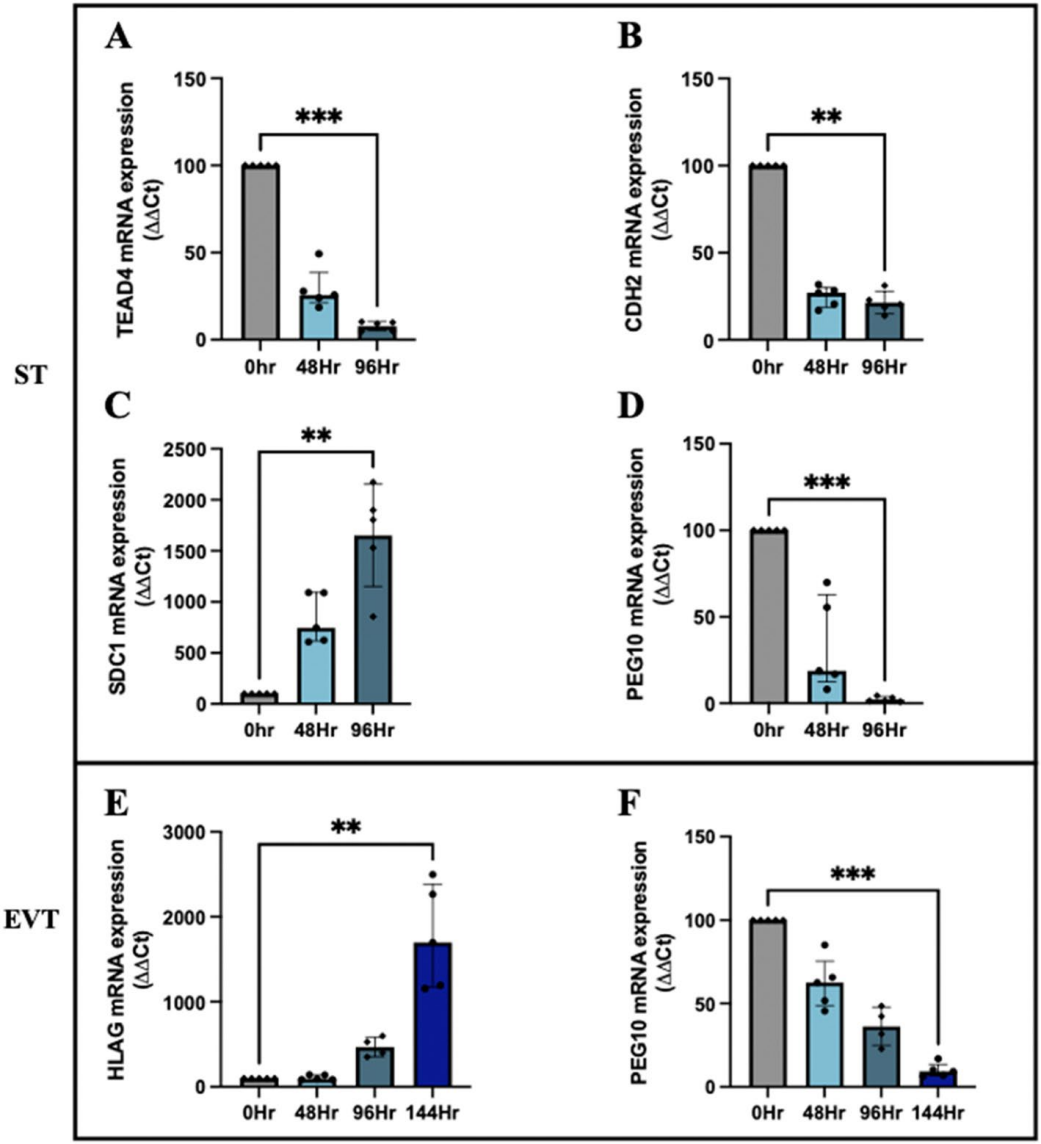
PEG10 gene expression reduces with cytotrophoblast differentiation. First-trimester human cytotrophoblast stem cells (hTSCs) were differentiated into syncytiotrophoblasts (ST) over 96 h (**A-D**)**.** Effective syncytialisation was confirmed by a decrease in trophoblast markers, *TEAD4* **(A)** and *CDH2* (**B**) and an increase in ST marker, *SDC1* (**C**). *PEG10* mRNA expression significantly decreased by 96 h in ST cells (**D**). hTSCs were differentiated into extravillous trophoblast (EVT) cells across 144 h as shown by an increase in EVT marker, *HLAG* (**E**). *PEG10* mRNA expression was significantly decreased after 144 h of EVT differentiation (**F**). Data expressed as a median with interquartile range. Individual symbols represent *n* = 5 experimental repeats. P** < 0.01, P*** < 0.001

To confirm *PEG10* is cytotrophoblast-specific, its expression was measured during differentiation of hTSCs, into syncytiotrophoblasts (ST) or extravillous trophoblasts (EVT). Successful syncytialisation was confirmed by a reduction in progenitor marker, *TEAD4 (*Fig. 3A; *P* = 0.0006) and cell adhesion marker, *CDH2* (Fig. 3B; *P* = 0.005), combined with upregulation of syncytiotrophoblast marker, *SDC1* (Fig. 3C; *P* = 0.001). *PEG10* expression decreased in syncytiotrophoblast cells 96 h post-differentiation (Fig. 3D; *P* = 0.0006). Differentiation into EVT over 144 h was confirmed by an increase in EVT marker, *HLAG* (Fig. 3E; *P* = 0.005). *PEG10* mRNA expression was reduced in EVT cells at 96 (*P* = 0.04) and 144 h post-differentiation (*P* = 0.0002; Fig. 3F). Thus, this data confirms that *PEG10* appears to be specific to undifferentiated hTSCs which are cytotrophoblast-like.

### Cytotrophoblast *PEG10* expression in response to hypoxia and inflammatory stimulation

**Fig. 4:**
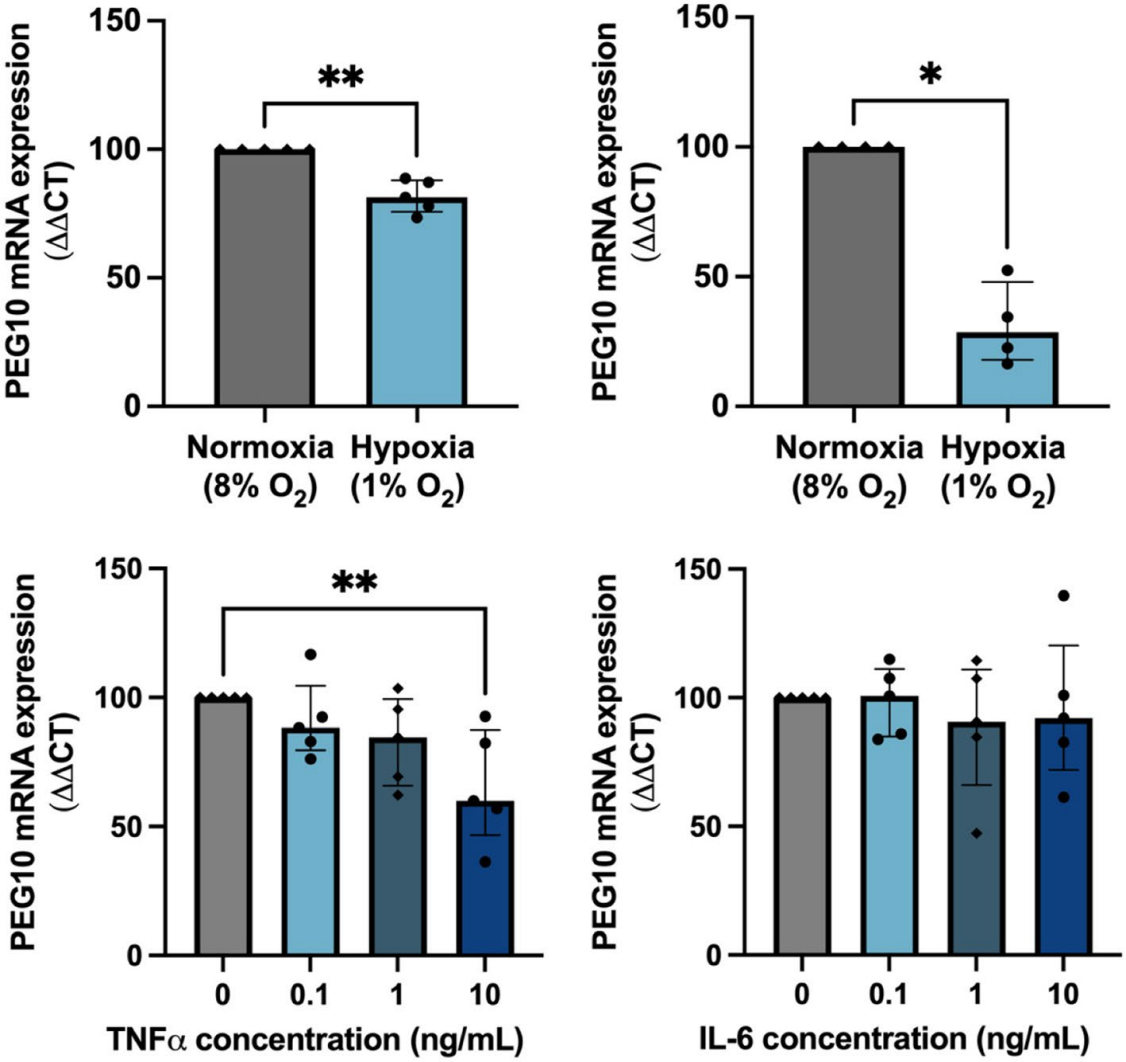
The effect of hypoxia and inflammatory cytokines on *PEG10* expression in cytotrophoblast stem cells. First-trimester human cytotrophoblast stem cells (hTSCs) or term primary cytotrophoblast were exposed to (**A-B**). *PEG10* mRNA expression was significantly reduced in hTSCs exposed to hypoxia (1% oxygen) relative to normoxia (8% oxygen) (**A**). A similar finding was observed when primary term trophoblast were exposed to hypoxia (**B**). Next, hTSCs were treated with increasing doses of TNFα (0, 0.1, 1 and 10 ng/mL) and showed a dose-dependent reduction in *PEG10* expression when compared to vehicle-treated controls (**C**). No changes in *PEG10* expression were observed in cells with increasing doses (0, 0.1, 1, 10 ng/mL) of IL-6 (**D**). Data is presented as a median with interquartile range. Individual symbols represent *n* = 5 experimental repeats and P** < 0.01

Preeclampsia pathology is associated with poor placental perfusion and intermittent hypoxia and inflammation [33, 34]. *PEG10* expression was measured in hTSCs in response to hypoxic (1% oxygen) and normoxic conditions (8% oxygen). *PEG10* mRNA expression was significantly reduced in hTSCs following hypoxia exposure (Fig. 4A; *P* = 0.008). We also measured levels in term cytotrophoblasts exposed to hypoxia (1% Oxygen) or normoxia (8% Oxygen), and similarly observed significantly reduced PEG10 mRNA expression (Fig. 4B, *p* < 0.05).

*PEG10* expression was also measured in hTSCs treated with either the inflammatory cytokine, tumour necrosis factor alpha (TNFα) or interleukin-6 (IL-6). Compared to vehicle controls, TNFα treatment significantly reduced *PEG10* mRNA expression at a concentration of 10 ng/mL (Fig. 4C; *P* = 0.03). No change in *PEG10* expression was observed in cells treated with IL-6 (Fig. 4CD).

### PEG10 knockdown does not alter trophoblast differentiation

**Fig. 5:**
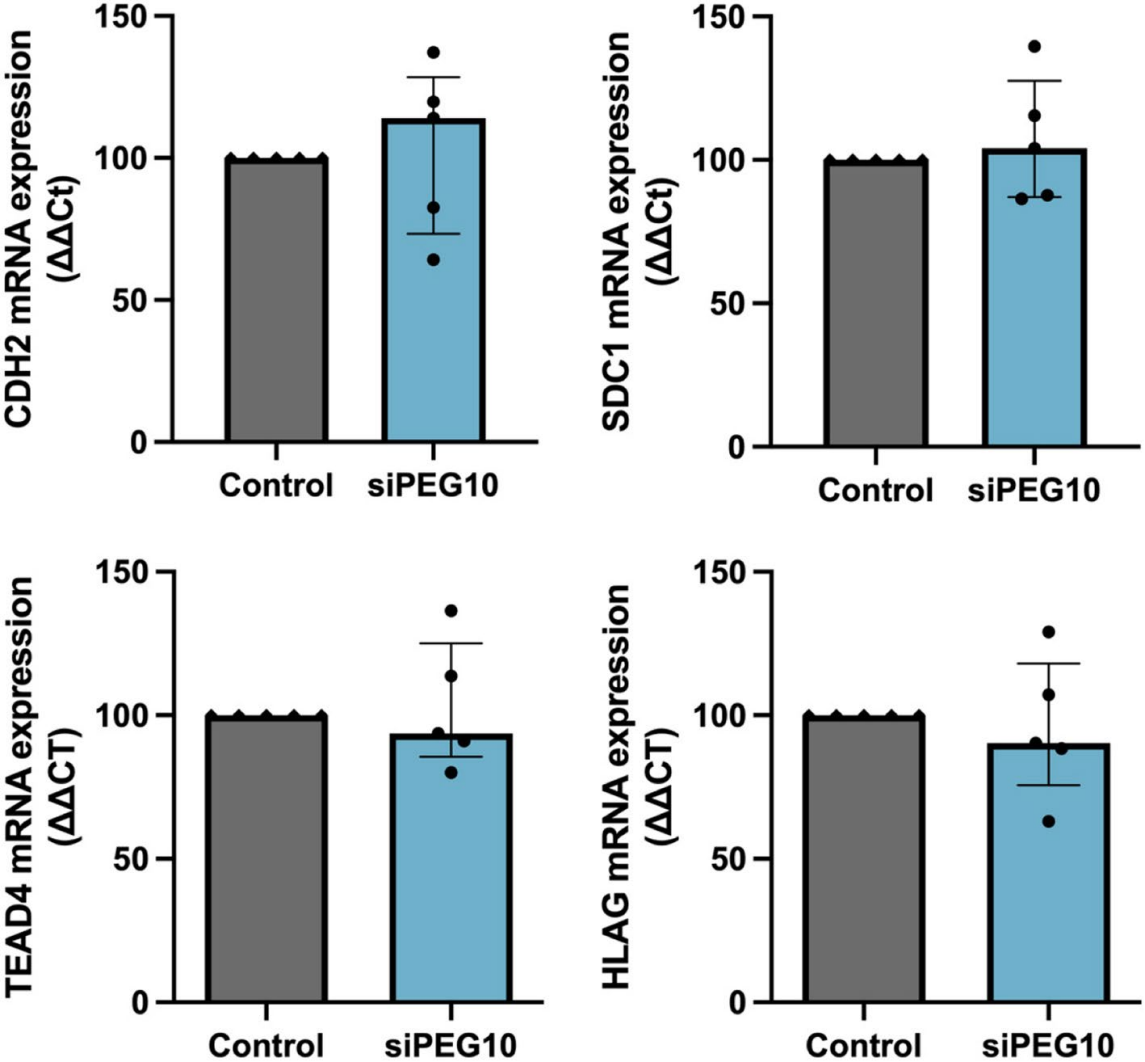
Effect of silenced PEG10 on trophoblast differentiation. First-trimester human cytotrophoblast stem cells (hTSCs) were treated with *PEG10* siRNA prior to differentiation into syncytiotrophoblasts over 96 h. No change in the expression of hTSC and syncytiotrophoblast markers, *CDH2* (**A**) and *SDC1* (**B**) were observed in *PEG10* siRNA-treated cells, relative to negative controls. Next, PEG10-silenced hTSCs were differentiated into EVTs over 144 h. No change in hTSC or EVT markers, *TEAD4* (**C**) and *HLAG* (**D**) were observed in *PEG10* siRNA-treated cells, compared to controls. Data expressed as a median with interquartile range. Individual symbols represent *n* = 5 experimental repeats

As PEG10 is predominately expressed in cytotrophoblasts, we assessed whether PEG10 is involved in trophoblast differentiation. However, in cells where PEG10 was reduced by siRNA treatment followed by differentiation, there was no change in syncytiotrophoblast differentiation markers, *CDH2* (Fig. 5A) and *SDC1* (Fig. 5B) compared to siRNA controls. Similarly, no change was observed in markers, *TEAD4* (Fig. 5C) and *HLAG* (Fig. 5D) in cells where PEG10 was reduced prior to EVT differentiation, compared to controls. Overall, this suggests reduced PEG10 does not affect trophoblast differentiation into syncytiotrophoblast or EVT.

### PEG10 knockdown impairs cytotrophoblast proliferation and canonical TGF $-{\varvec{\beta}}$ signalling

**Fig. 6:**
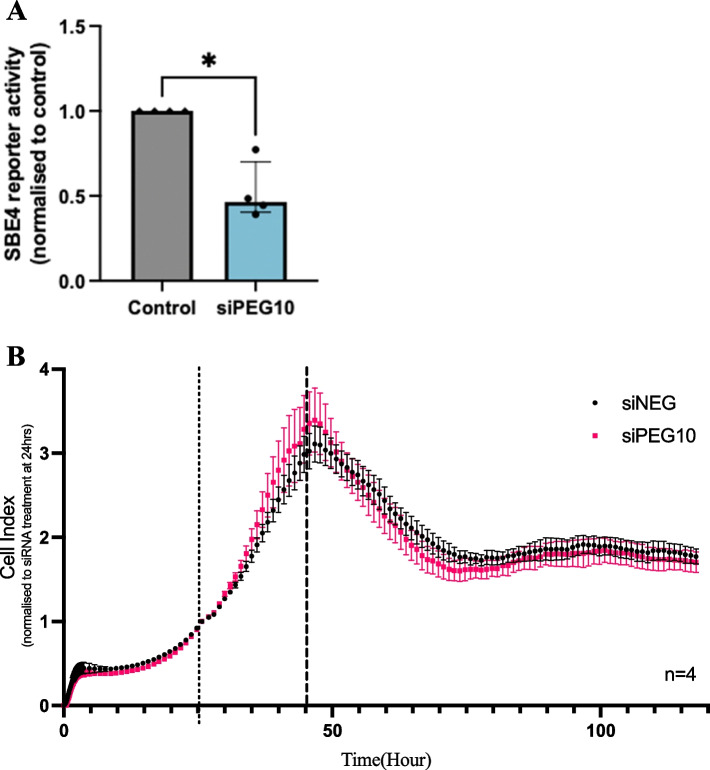
Effect of silenced *PEG10* on cytotrophoblast function. Following transfection with luciferase reporter plasmid containing four copies of the SMAD binding element, *PEG10*-siRNA hTSCs had reduced basal levels of canonical TGF $-\beta$ signalling relative to negative siRNA controls (**A**). Real-time analysis of cell proliferation showed no effect of PEG10 siRNA (pink) relative to control siRNA treated cells (black) (**B**). Dotted line in panel B indicates time of siRNA treatment, dashed line indicates time of media change. Data expressed as median with interquartile range. P* < 0.05

Next, we measured the effect of reduced PEG10 on canonical TGF $-\beta$ signalling in cytotrophoblast cells. A luciferase reporter plasmid (containing four copies of the SMAD binding element) was co-transfected into *PEG10* siRNA-treated cytotrophoblasts. Luciferase activity in PEG10 siRNA-treated cytotrophoblasts showed a reduction in basal canonical TGF $-\beta$ signalling, relative to negative siRNA-treated controls (Fig. 6A, P = 0.03). As TGF $-\beta$ signalling has been associated with cell growth, we next measured hTSC proliferation following PEG10 knockdown using siRNA. Real-time analysis using the xCELLigence system showed no effect on proliferation of hTSCs following PEG10-siRNA treatment (Fig. 6B).

## Discussion

This study identified that PEG10 gene and protein expression are decreased in placentas from patients with early-onset preeclampsia. Immunohistochemistry and in situ hybridisation experiments demonstrated cytotrophoblasts were the primary cell type expressing PEG10. Further in vitro studies found that hypoxia and inflammation decreased *PEG10* in hTSCs. Functional studies of reduced PEG10 in hTSCs showed reduced activation of the SMAD binding element, suggesting a relationship with the TGF-$\beta$ signaling pathway in the placenta.

PEG10 is critical in placental development, with mouse knockout models demonstrating early embryonic lethality due to impaired placentation [16]. Our study showed that PEG10 gene and protein expression is decreased in placenta from patients with early-onset preeclampsia. To date, only two studies have characterised PEG10 in preeclampsia and the published data is inconsistent. Chen et al*.* found increased PEG10 gene and protein expression in late-onset preeclampsia, defined as onset $\ge$ 34 weeks’ gestation which differs in aetiology and severity compared to the early-onset type [27, 35]. Early-onset preeclampsia has been associated with impaired placental spiral artery remodelling whereas placentas from late-onset preeclampsia patients show fewer placental defects. Indeed, late-onset preeclampsia is often attributed to maternal factors such as higher BMI or familial hypertension [36, 37]. Transcriptome studies have identified different molecular signatures between early and late-onset preeclampsia so it is possible that PEG10 may be differentially expressed in both [38, 39]. Another study by Liang et al*. *[28] demonstrated decreased PEG10 gene and protein in preeclamptic placentas, but their samples (mean gestational age 34.2 $\pm$ 3.3 weeks) were not well-matched with controls (mean gestational age 39.1 $\pm$ 0.7 weeks). The strengths of the current study include the use of a large, carefully characterised cohort of samples including gestation-matched controls (mean gestational age early-onset preeclampsia, 29.65 $\pm$ 0.27 weeks; control, 30.25 $\pm$ 0.59 weeks). It should be noted that PEG10 protein measurements in the current study and those previously mentioned by Liang et al*.* and Chen et al*.* are all based on semi-quantitative analysis of immunohistochemistry staining and should be validated in a large cohort with a more quantitative measure. Due to difficulties measuring PEG10 protein within our cohort, our functional studies largely focused on gene expression changes.

Placental hypoxia and inflammation are hallmarks of preeclampsia, and our in vitro results show these downregulate *PEG10* expression. Decreased *PEG10* mRNA expression was observed in hTSCs following exposure to hypoxia (1% O_2_). Smallwood et al*.* showed that PEG10 expression was low during the hypoxic phase in early pregnancy but increases at 11–12 weeks’ gestation [40]. PEG10 upregulation occurs just prior to the onset of maternal blood flow to the placenta at 12–13 weeks’ gestation, following complete trophoblast remodelling of spiral arteries [41]. It is likely that PEG10 upregulation has critical functions pertaining to this period of placental development at the conclusion of the first trimester [42]. In early onset preeclampsia, poor perfusion due to impaired spiral artery remodelling results in prolonged exposure of the placenta to intermittent hypoxia [43]. While the effect on *PEG10* in hTSCs exposed to hypoxia may recapitulate the changes observed across the first trimester, it is also possible that the hypoxia present in preeclamptic placentas may contribute to reduced PEG10. To test this hypothesis, we also exposed isolated term cytotrophoblast to normoxia (8% O_2_) or hypoxia (1% O_2_), and similarly observed a significant reduction in *PEG10* mRNA expression.

Our study also identified that *PEG10* was downregulated in hTSCs exposed to the pro-inflammatory cytokine, TNF $\alpha$ but not IL-6. These cytokines are of interest as they are markedly increased in placentas from preeclamptic pregnancies [34]. No prior literature exists on interactions between PEG10 and TNF $\alpha$ nor IL-6. However, interactions between TNF $\alpha$ and TGF-$\beta$ have been reported [44] and TGF-$\beta 1$ has been shown to inhibit PEG10 expression [45]. A positive correlation between TNF $\alpha$ and TGF-$\beta 1$ has been demonstrated in fibroblasts [46], thyroid cells [47] and placental mesenchymal stem cells [48]. Thus, increased TGF-$\beta$ signalling may account for PEG10 downregulation in response to TNF $\alpha$ but not IL-6. However, preeclampsia is marked by complex immune modulation and singular cytokines are not wholly representative of the disease state [49]. Further studies could elucidate whether a longer exposure of cytokines such as IL-6 or a combination of inflammatory factors further dysregulate PEG10 expression.

The work herein identified PEG10 as a molecule expressed in the cytotrophoblast. This was demonstrated using both immunohistochemistry and in situ hybridisation. We further validated findings that PEG10 is specific to this progenitor cell type by showing *PEG10* was downregulated during hTSC differentiation into syncytiotrophoblasts and extravillous trophoblasts. It should be noted that *PEG10* is still detectable in these two differentiated populations, but expression is significantly lower than in hTSCs. This concurs with prior findings that PEG10 is expressed in all three equivalent trophoblast layers in the mouse and human placenta [16, 50].

Our results show that PEG10 knockdown was associated with reduced activation of the SMAD binding element (SBE) which regulates canonical TGF-β target gene transcription. This is an interesting find, give the hTSCs are grown with ALK4, 5 and 7 inhibitors, key receptors of the TGF-β signalling pathway. While PEG10 is not a direct effector of TGF-$\beta$ signalling, this data suggests it may be involved in regulation of this pathway in trophoblasts. The TGF-β signalling pathway has critical functions in early gestation including placental trophoblast proliferation, differentiation, and migration [51, 52]. Previous studies have shown an interaction between PEG10 and both the canonical and non-canonical TGF-$\beta$ signaling pathways in cancer cells [18, 25, 53, 54]. Akamatsu et al*.* also measured SBE reporter activity in a highly metastatic adenocarcinoma cell line and showed reduced canonical TGF-$\beta$ signaling in PEG10 silenced cells [25]. This concurs with the results of our study, which is the first to show this association between PEG10 and canonical TGF-$\beta$ signaling in placental cells. Further investigation regarding the mechanism of action for reduced cellular activation of the canonical TGF-$\beta$ effector-SBE due to PEG10-silencing is required to validate these findings. Although the TGF-$\beta$ superfamily has been shown to regulate trophoblast proliferation [55], our study did not show any effects of PEG10-silencing on hTSC proliferation in xCELLigence assays. This highlights a limitation of using the hTSCs, whereby the presence of ALK inhibitors that assist in maintaining their stem-like capacity might impair insights into the pathways that utilise these super highways. As such, future studies should consider inhibiting PEG10 in primary first trimester cytotrophoblast, alongside alternate approaches to reducing PEG10 such as utilising shRNA or utilising the CRISPR-Cas system. This will allow insights into whether longer-term silencing of PEG10 in the absence of pathway inhibitors reveals more pronounced effects on the TGF-$\beta$ signalling pathway and possibly cytotrophoblast differentiation/proliferation.

## Conclusions

This study provides strong evidence that PEG10 is reduced in placenta from preeclamptic pregnancies and identified that placental hypoxia or inflammation may contribute to this. In vitro studies showed that reduced PEG10 decreases TGF-$\beta$ signalling but does not appear to impair trophoblast differentiation or proliferation. This study provides important insights into the dysregulation of PEG10 in placentas from preeclamptic pregnancies and provides avenues for further investigation into the functional relevance of this reduction.

## Data Availability

The dataset used is available from the corresponding author upon reasonable request.

## Abbreviations

ACOG: American College of Obstetricians and Gynaecologists
CDH2: N-cadherin 2
cDNA: Complementary deoxyribonucleic acid
Cq: Quantification cycle
Ct: Cycle threshold
CYC1: Cytochrome C1
dPBS: Dulbecco’s phosphate buffered saline
ELISA: Enzyme-linked immunosorbent assay
EVT: Extravillous trophoblast
GAPDH: Glyceraldehyde 3-phosphate dehydrogenase
GATA3: GATA binding protein 3
HLA-G: Human leukocyte antigen G
hTSC: Human (cyto)trophoblast stem cell
IHC: Immunohistochemistry
IL-6: Interleukin-6
mRNA: Messenger ribonucleic acid
PECAM-1: Platelet endothelial cell adhesion molecule
PEG10: Paternal expressed gene 10
RNA: Ribonucleic acid
RT-PCR: Reverse transcriptase polymerase chain reaction
SBE: SMAD binding element
SDC1: Syndecan-1
SIRH: Sushi-ichi-related retrotransposon homolog
siRNA: Short interfering ribonucleic acid
ST: Syncytiotrophoblast
TEAD4: TEA domain transcription factor 4
TNFα: Tumour necrosis factor α
TGF-β: Transforming Growth Factor beta
